# Infantile Diphtheria: A Special Danger in the First Year of Life

**Published:** 1916-08-12

**Authors:** 


### INFANTILE DIPHTHERIA.

### A Special Danger in the First Year of Life.

It has long been familiar to clinicians and duly emphasised in text-books that diphtheritic infection, though uncommon in infants under a year old, is especially deadly to those of them that it does happen to attack. These and other peculiarities of the disease in young infants have attracted the attention of many observers at different times; the latest contribution to the subject, an exceptionally valuable one, is that published recently by Dr. J. D. Rolleston, a well-known London expert on infectious fevers in general and on diphtheria in particular.*

### Partial Immunity of Babies.

The percentage of diphtheria patients under one year old admitted to the Metropolitan Asylums Board hospitals during the last fifteen years has varied between 1.5 per cent. and 2.8 per cent. annually of the total cases admitted. Continental statistics show somewhat higher proportions of infants among diphtheria patients: thus figures from Berlin have been as high as 5.5 per cent., and from Russia a percentage of 4 has been reported. In the last 2,600 cases which have passed through Dr. Rolleston's hands, however, there have been only twenty, or less than 1 per cent., in the first year of life. The majority of the cases occurred during the seventh to twelfth months, and this is in accordance with the experience of other observers. Various suppositions have been advanced to explain the comparative rarity of diphtheria in babies. "It has been variously attributed," says Dr. Rolleston, "to immunity acquired *in utero*, to antitoxin transmitted through the mother's milk, to the acid reaction of the secretions of the buccal cavity of the infant, which is inimical to the growth of the bacilli, and to the greater care and comparative isolation which the nursling as a rule enjoys." All authorities are agreed that it is commoner in the artificially fed, but breast-nursed children are not immune. The youngest patients on record are probably those of Auden at eight days, and of Kühn at two weeks: the latter was a nasal case, the former was of the usual faucial type.

### The Heavy Mortality.

Although, as has been shown, the morbidity rates for these quite young children are low, the mortality, on the other hand, has always been high. The Asylums Board statistics show annual rates ranging from 14 to 41.4 per cent. of deaths, with an average of 32.1, whereas for all ages the extreme rates have been 6.5 and 12.5 per cent. These figures refer, of course, to the antitoxin period: before then the Asylums Board lost 70 per cent. of those under a year and 31 per cent. at all ages. The mortality is always heaviest in those cases that require tracheotomy or intubation. Before antitoxin came in recovery was very unusual after either of these operations had been performed on an infant. Between 1884 and 1894 thirty-seven children under a year were tracheotomised at the Berlin University Surgical Clinic; not a single one survived. Still, there were occasional successes, even without antitoxin, in those cases. Nowadays the mortality rate among children under a year submitted to tracheotomy or intubation varies from about 45 to 60 per cent.; and, in addition, many of them who without antitoxin would have to be thus operated upon, never require interference and get perfectly well on antitoxin alone.

The reasons for this severe mortality rate in infancy include, no doubt, the extra liability to respiratory complications and their unusual gravity when the latter occur; and the liability to mechanical obstruction to the narrow larynx by a membrane which would not obstruct the air entry of an older child. But there must also, besides, be some especial vulnerability to the diphtheritic toxins, one would think, to explain the exceedingly heavy death-rate in these little people. If this is the case, it tells against some of the hypotheses put forward to explain the rarity of infection; those, namely, that presuppose some antitoxic properties in the mother's milk.

### The Distribution of the Lesions.

In Dr. Rolleston's twenty cases there were five that were purely faucial, six that were purely nasal, and one that was purely laryngeal. In eight there were multiple lesions, and it is remarkable that in three of these there was cutaneous affection. This frequency of 15 per cent. of cutaneous diphtheria contrasts with 1.1 per cent. for the total 2,600 cases. The explanation of this phenomenon seems obscure, unless it is that infants are more liable to pick their noses, and so to spread the bacilli thence to other places. Curiously enough, there was no case of conjunctival diphtheria in Dr. Rolleston's series: this form is not so very rare, and is almost confined to little children, so one would have expected to find it represented.

Not only was the infection purely nasal in six cases, there were five others in which the nose was involved together with another region. So that 65 per cent. of the cases presented diphtheria of the nasal passages, as against a percentage of 25.6 for the series at all ages. It is curious that in every breast-fed baby except one among Dr. Rolleston's cases the infection was confined to the nose. The larynx became involved in four cases; one case required tracheotomy and ended fatally, the other three did well.

Many authorities have emphasised the importance of congenital syphilis as a predisposing cause of diphtheria in the infant. This, it is suggested, may be due not merely to a weakening of the general constitutional resistance to infection, but also by the common syphilitic rhinitis, rendering the nasal fossæ especially susceptible to attack.

* *American Journal of Diseases of Children*, July, 1916, pp. 47-52.

Three of Dr. Rolleston's patients were obviously syphilitic, and two of them died, though their diphtheria was of quite a mild character. He believes that the Wassermann reaction would have revealed a higher percentage still of luetic infection, and that the two children in question died more of syphilis than of diphtheria. The most experienced authorities on diphtheria all lay stress on this extra frequency among infants suffering from syphilis, a relation which has been rendered much more striking statistically by the results of Wassermann tests.

### Paralysis.

Diphtheritic paralysis is rarer among young children than at older ages. Possibly this may be due to the high mortality rate, which sweeps off many of the severer cases, in which, had they recovered, diphtheritic paralysis might subsequently have developed. Only two cases were noted by Dr. Rolleston, in both of which there was an early invasion of palatal and cardiac palsy in very severe faucial diphtheria—the incidence of paralysis at all ages was twice as high—namely, 19.8 per cent. On the other hand, broncho-pneumonia, which supervened in but 1.2 per cent. of the 2,600 cases, was present in six of the twenty infants, five of whom died of it. Besides these five deaths from pneumonia there were two from cardiac paralysis and two from syphilis, or nine in all. The fatality rate was thus 45 per cent. of the cases.

### Treatment.

Dr. J. D. Rolleston is one of those who favour very large doses of serum, which he often gives intramuscularly instead of subcutaneously. In a mild attack he gives 1,000 to 4,000 units; for moderate attacks 8,000 to 12,000; and for severe attacks 12,000 to 24,000, repeated if necessary. The highest total reached in any case of this series was 42,000 units; the patient died. Those extraordinary people whose obsession by a fad prevents their appreciation of the overwhelming testimony in favour of antitoxin treatment, simply because it is based upon vivisection, may possibly argue that it was the serum, not the diphtheria, that killed the infant. All one can say to that is that the large doses of serum were given because the case was of very exceptional severity; that some of the other cases that had doses almost equally large pulled through; and that the two patients who received the smallest doses of the whole series both died. Dr. Rolleston expresses no opinion on the relative merits of tracheotomy and of intubation for these tiny patients; except for the very unusually expert intubator, probably tracheotomy is the safer at this age, and the more satisfactory in its results.

